# Bronchoalveolar Lavage Results Are Independent of Season, Age, Gender and Collection Site

**DOI:** 10.1371/journal.pone.0043644

**Published:** 2012-08-31

**Authors:** Helga H. Olsen, Johan Grunewald, Göran Tornling, C. Magnus Sköld, Anders Eklund

**Affiliations:** Department of Medicine, Division of Respiratory Medicine, Karolinska University Hospital, Solna, Stockholm, Sweden; University Hospital Freiburg, Germany

## Abstract

**Background:**

Clinical interpretation of bronchoalveolar lavage fluid results is dependent on the availability of reference values for healthy individuals. Only a few studies have published such reference values and the applicability of results is restricted by small sample sizes and the limited representativeness of the study population. We aim to investigate the influence of age, gender, collection site and season on bronchoalveolar lavage fluid results and to establish reference values for use in clinical practice.

**Methodology/Principal Findings:**

Bronchoalveolar lavage fluid data from 295 healthy never-smoking volunteers, investigated during 1990–2009, were analyzed retrospectively. 47 volunteers had 2–5 repeat lavages during the course of several years. Fluid recovery, total number of cells, cell concentration, and differential cell counts on cytospin prepared slides were recorded. Reference values, as represented by the 5^th^ to the 95^th^ percentile, were 72–96% for macrophages, 2–26% for lymphocytes, 0–4% for neutrophils and 0–1% for eosinophils. Basophils and mast cells were rare. When repeat lavages were performed, there was a relatively large intra-individual variability, mainly for macrophages and lymphocytes. An age dependent decrease of lavage fluid return was present, but there was no age dependent correlation with any of the other BALF parameters. The BALF cell parameters were independent of gender, season and site (lingula vs. middle lobe).

**Conclusions/Significance:**

Our data show that bronchoalveolar lavage fluid cell differential count is independent of age, gender, season and collection site (RML or lingua). It therefore seems acceptable to use the same reference values for all never-smoking individuals.

## Introduction

Bronchoalveolar lavage (BAL) allows sampling of cells and non-cellular components of the epithelial lining fluid. It is easily available, minimally invasive and generally safe [1]. The collection of BAL fluid (BALF) has proven an invaluable tool both in clinical practice and in research, as it aids the diagnoses of various pulmonary diseases and provides insights into the disease mechanisms.

The clinical application of BALF analysis requires a standardized procedure for the collection and processing of BALF, as well as representative reference values. Recommendations for performing and analyzing BAL have been published by both the British Thoracic Society and the European Respiratory Society [2], [3]. Some studies have addressed the issue of normality for BALF return volume and differential cell counts from healthy, never-smoking adults [4]–[17] (Table 1). Recently the American Thoracic Society published a guideline for the clinical utility of bronchoalveolar lavage cellular analysis in interstitial lung disease [18]. Based on 7 studies [4], [6], [9], [13], [15]–[17] including a total of 327 never smoking and 175 non-smoking healthy volunteers, normal BAL cellular patterns were specified as follows: Alveolar macrophages >85%, lymphocytes 10–15%, neutrophils ≤3%, eosinophils ≤1% and mast cells ≤0.5%. A similar review was performed by Balbi et al [19] who identified 9 studies looking at BALF parameters in healthy volunteers [4]–[12]. In these studies a total of 760 subjects were included, of which 478 subjects were never-smokers. The upper cut-off points for the differential cell counts (mean+2SD) in the never-smokers from these 9 studies were 16.7% for lymphocytes, 2.3% for neutrophils and 1.9% for eosinophils.

**Table 1:** Reference values for bronchoalveolar lavage fluid findings in healthy non-smoking and never smoking subjects from previously published studies.

REF	Country	N	Smoking	F (%)	Instilled fluid (ml)	Site	Age (Years)	Recovery (%)	Cell conc (×10^3^/ml)	Macrophages (%)	Lymphocytes (%)	Neutrophils (%)	Eosinophils (%)
**4**	The US	77	Never	51	60×4	RML	40 (*13*)	*64* (*14.6*)	129	85.2 (*14.0*)	11.8 (*9.7*)	1.6 (*0.6*)	0.2 (*0.5*)
**15**	The US	38	Never	NA	60×4	RML	[18–40]	69 (*6.2*)	105	89.5 (*6.8*)	9.2 (*6.8*)	1.0 (*1.2*)	0.1 (*0.6*)
**15**	The US	30	Never	NA	60×4	RML	[≥65]	54 (*11*)	158 (*93.1*)	80.2 (*11.5*)	15.1 (*11.5*)	4.3 (*4.9*)	0.5 (*1.1*)
**15**	The US	23	Never	NA	40×4	RML	[18–40]	67.5 (*4.8*)	103 (*43.2*)	88.7 (*5.8*)	9.4 (*5.8*)	1.4 (*0.5*)	0.3 (*0.5*)
**15**	The US	20	Never	NA	40×4	RUL	[18–40]	60.1 (*8.9*)	114 (*58.1*)	88.9 (*6.3*)	9.0 (*6.3*)	1.9 (*2.2*)	0.2 (*0.4*)
**13**	Finland	18	Never	44	20×10	RML	33.4 (8.3)	86.0 (7.3)	107.7 (70.3)	85.3 (9.0)	12.6 (8.6)	1.7 (2.1)	0.35 (0.62)
**16**	The Netherlands	28	Non	43	50×4	RML	39 [19–60]	58.4 (*14.8*)	103 (*79*)	89.8 (*3.7*)	8.4 (*3.7*)	1.3 (*1.0*)	0.44 (*0.5*)
**6**	The US	78	Non	44	40×3	Lingula	26.3 (3.6) [20–36]	63.4 (10.8) [23.3–79.2]	93.7 (45.1) [9.9–267]	95.1 (2.9) [84.2–99.3]	3.9 (2.4) [0.7–14.4]	0.7 (0.8)	0.17 (0.9) [0–7.1]
**17**	The US	19	Non	53	50×6	RML	25.7 (*7.4*) [18–41]	70.5 (*8.7*) [53.0–86.1]	116 (*69.7*) [64–375]	*91* (*2.6*)	*8.3* (*3.9*)	*0.8* (*2.6*)	*0.3* (*0.9*)
**9**	The US	111	Never	37	5×20	RML/Lingula	30 [20–48]	*76*	127 (91) [20–840]	93.2 (5.8) [61–100]	6.1 (5.6) [0–38]	0.54 (0.83) [0–5]	0.14 (0.47) [0–3]
**5**	Sweden	17	Never	0	3×50	RML	60 [60–60]	59 [33–80]	92 [25–200]	87 [75–96]	10 [0–23]	2 [1–5]	0.8 [0–3]
**8**	Canada	42	Non	48	10×30 (37)/5×20 (5)	RML	24.8 [19–41]	69.0 (7.3) [45–82.7]	58 [18–144]	88.6 (7.9) [60–98.7]	9.6 (7.7) [1–39.8]	1.7 (1.2) [0–4.6)	NA
**10**	The US	15	Never	47	4×60	NA	28.1 (*5.0*) [20–36]	*68.5* (*9.0*)	89.5 (*35.6*)	85.8 (*7.7*)	12.4 (*6.6*)	1.84 (*1.01*)	0.11 (*0.31*)
**10**	The US	15	Never	47	4×60	NA	69.3 (*3.9*) [≥65]	*54.6* (*10.2*)	141.9 (*73.6*)	81.5 (*10.5*)	13.2 (*10.1*)	4.61 (*4.76*)	0.71 (*1.47*)
**11**	The US	19	Never	37	4×60	RML	27 (*4*) [19–36]	*67.9* (*3.6*)	122 (*39*)	90 (*4*)	8.3 (*3.9*)	1.2 (*0.9*)	0.3 (*0.4*)
**11**	The US	15	Never	53	4×60	RML	71 (*4*) [64–83]	*56.3* (*12.9*)	163 (*97*)	80 (*12*)	17.0 (*12.8*)	2.7 (*2.7*)	0.3 (*0.4*)
**12**	The US	24	Non	NA	250	NA	NA	*64* (*10*)	*139* (*104*)	78.8 (*18.6*)	16.7 (*14.7*)	NA	NA
**This study**	Sweden	295	Never	55	5×50	RML	31.5 (*11.7*) [18–65]	71.9 (9.4) [42–90]	91.9 (41.7) [29.3–370]	88.1 (8.2) [50.2–98.2]	9.6 (7.7) [0.8–48.2]	1.85 (1.96) [0–18.30]	0.29 (0.63) [0–6.0)

Results reported in ≤10 subjects were excluded from the table.

Definition of abbreviations: REF = references, N = number, F = female, NA = not available, Cell conc = cell concentration.

Values are Mean (SD) [Range].

Numbers in italic are calculated from the publication.

However, the interpretability of results from these previous studies is limited by the small sample sizes, with only 18–138 non-smoking and never smoking subjects in the individual studies. In addition there were large differences in methodology across trials, and the majority of participants were young to middle aged men, also narrowing the applicability of results. Regarding intra individual reproducibility of BALF results the data is even more limited [20]–[22].

Furthermore, healthy volunteers are heterogeneous, including individuals of different age, gender, ethnicity and lifestyle. Smokers have an increased total cell count, mainly due to an increased proportion of macrophages [23]. Older subjects seem to have a lower total volume of retrieved fluid and they may have an increased proportion of neutrophils, and/or lymphocytes [10], [11]. These apparent differences in BALF composition, poses the question of whether or not it would be beneficial to have separate reference values for individual subgroups, such as for subjects of different age and gender. Furthermore, little information is available regarding whether BALF composition varies depending on the location where it is collected (lingula vs middle lobe) and if there is a seasonal variation in the BALF constituents. In this article we report the results of BALF analysis from a large group of healthy never-smoking volunteers to address the questions above.

## Materials and Methods

### Objectives

In this study, we aim to investigate the influence of age, gender, collection site and season on bronchoalveolar lavage fluid results in healthy volunteers and to establish reference values for use in clinical practice.

### Participants

We performed a retrospective analysis of BALF findings in healthy never-smoking volunteers investigated at the Karolinska University Hospital in Stockholm, Sweden, from 1990 to 2009. The individuals were recruited by word of mouth and by advertisement and were reimbursed for their participation. Two hundred and ninety five subjects aged 18–65 years (163 women (mean age 31.4 years, SD 11.5) and 132 men (mean age 31.7 years, SD 11.9)) were included. The subjects had no respiratory symptoms, normal cardiopulmonary exam, no past medical history of respiratory disease, took no medications and had no history of viral- or other illness at least one month prior to the bronchoscopy. They were all living in an urban environment (Stockholm area). To ensure standardization a few, select investigators performed all consultations and examinations. Fluid recovery, total number of cells, cell concentration, and differential cell counts were recorded, as were the BALF collection site and the date of the bronchoscopy.

### Bronchoscopy and BAL

All BAL samples were collected and analyzed according to a standardized procedure [24]. A limited number of investigators performed all bronchoscopies, ensuring methodological homogeneity. Bronchoscopies were performed in the morning on an outpatient basis. Patients were nil by mouth for at least 6 hours prior to the procedure. Premedication with intramuscular morphine-scopolamine was given 45 minutes prior to the investigation. Individuals that could not tolerate morphine-scopolamine, received intramuscular pethidine and subcutaneous atropine. Bronchoscopies were carried out in the supine position with a flexible fiberoptic bronchoscope (Olympus Optical Co. Ltd, Tokyo, Japan) inserted nasally. Lignocaine (Xylocain ®; AstraZeneca, Södertalje, Sweden) was used for local anesthesia. The bronchoscope was wedged in a subsegmental bronchus in the middle lobe or the lingua lobe. Five aliquots of 50 ml of sterile, phosphate-buffered saline solution at 37°C were instilled. After each instillation the fluid was gently aspirated with a negative pressure of −40–−50 mm Hg, adjusted to −10–−20 mm Hg if BALF recovery was poor. The fluid was pooled, collected in a siliconized plastic bottle kept on ice and immediately transported to the laboratory.

### Preparation of BALF cells

All BAL samples were prepared and analyzed at the Karolinska University Hospital Lung Research Laboratory. The BALF was strained through a single layer of Dacron net (Type AP32; Millipore, Cork, Ireland) and the volume of recovered fluid was measured. Recovery was expressed both as an absolute volume and as a percentage of instilled fluid. The fluid was centrifuged at 400×g for ten minutes at 4°C and the supernatant was removed. The cell pellet was resuspended in RPMI 1640 (Sigma). Total cell counts and assessment of viability by trypane blue cell exclusion were performed using a Bürker chamber (Marienfeld, Germany). Smears for differential cell counts were prepared by cyto-centrifugation (Cytospin 2; Shanon Ltd, Runcorn, UK) at 22×g for three minutes and stained with May-Grünwald Giemsa. 500 cells were counted. Mast cells in 10 visual fields (16× magnification) were determined after staining with toluidine/haematoxylin.

### Ethics

The study was approved by the Regional Ethics Committee in Stockholm. All subjects gave written and verbal informed consent.

### Statistical methods

Descriptive statistics were used to define the reference values, which were defined as the 5^th^ and 95^th^ percentiles of all included subjects. Comparisons between groups were performed by analysis of variance, using the Satterthwaite approximation in case of unequal variance between the groups. Correlation between BALF variables and age were analyzed by the Pearson correlation coefficient. Since the study was regarded as exploratory, no corrections due to multiple analyses were performed in order to avoid false negative conclusions, and a p value <0.05 was considered significant. However, p-values above 0.005 were interpreted with caution. In case that the subject had underwent more than one BAL, only the latest investigation was used in defining the reference values, comparison between groups and distribution of parameters over the calendar year. The within subject variability for BALF parameters was calculated by multiplying the mean intra-individual standard deviations with 1.96.

## Results

### Recovery, cell count and differential cell count

The results for the entire group of subjects are presented in Table 2. The quantity of fluid recovered ranged from minimum 42% to maximum 90%. The viability of BALF cells was 93% (median), with a range from 72–99%. There was considerable variability in the total cell numbers and the cell concentrations (median 85×10^6^/L, min-max 29–370×10^6^/L). The majority of recovered cells were alveolar macrophages (median 91%, min-max 50–98%). The median percentage of lymphocytes was 7 with a min-max of 0.8–48.2%. The reference value for lymphocytes as defined by the 5^th^ to the 95^th^ percentile was 2–26%. Approximately 10% of the study population had a percentage of lymphocytes greater than 20. Neutrophils and eosinophils were present in low numbers, although isolated higher values occurred in a few individuals. Six of the 295 subjects had a neutrophil count over 6, while only three individuals had a neutrophil count over 10. Basophils and mast cells were rare.

**Table 2:** Descriptive statistics of bronchoalveolar lavage fluid findings in healthy never-smoking subjects.

Variable	N	Minimum	Median	Maximum	5^th^ Pctl	10^th^ Pctl	90^th^ Pctl	95^th^ Pctl
**Age** (years)	295	18	27	65	19	21	51	59
**Sex** (females/males)	163/132							
**Return volume** (mL)	266	106	184	226	132	149	206	210
**Recovery** (%)	266	42	74	90	53	60	82	84
**Viability** (%)	292	72	93	99	82	85	98	98
**Total Cell Number** (×10^6^)	266	5	16	74	8	9	24	28
**Cell concentration** (×10^6^/L)	266	29	85	370	43	51	142	171
**Macrophages** (%)	284	50	91	98	72	76	96	96
**Macrophages** (×10^6^/L)	255	23	74	248	36	44	117	157
**Lymphocytes** (%)	284	0.8	7	48.2	2	3	20	26
**Lymphocytes** (×10^6^/L)	255	0.4	6	178.3	2	2	19	24
**Neutrophils** (%)	284	0	1	18.30	0	0	3	4
**Neutrophils** (×10^6^/L)	255	0	1	16.31	0	0	3	4
**Eosinophils** (%)	283	0	0	6.00	0	0	0	1
**Eosinophils** (×10^6^/L)	254	0	0	5.10	0	0	0	1
**Basophils** (%)	284	0	0	0.40	0	0	0	0
**Basophil** (×10^6^/L)	255	0	0	0.35	0	0	0	0
**Mast Cells** (per 10 visual fields)	214	0	1	48.00	0	0	7	10

### Correlation with age and gender for BALF results

**Figure 1:**
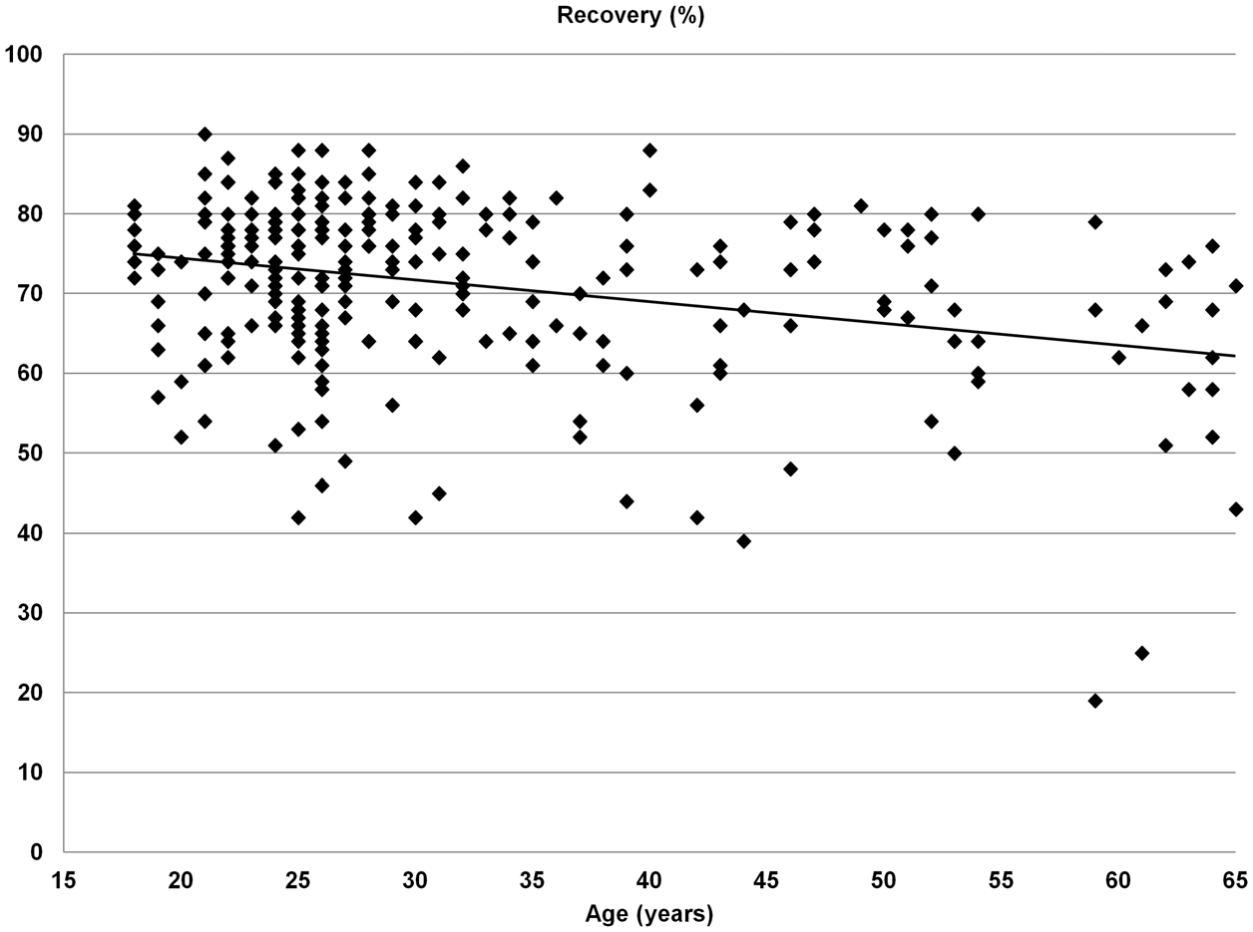
Correlation with age for bronchoalveolar lavage fluid recovery (y-axis) in healthy never-smoking subjects.

Table 3 demonstrates the correlation with age for bronchoalveolar lavage findings in healthy never-smoking subjects. Although relatively small, there was a statistically significant age related decline in the volume of BALF recovered (p<.0001) (Figure 1). None of the other parameters were age dependent. However, a few individuals had an eosinophil count that fell outside the reference range as determined by the 5^th^ to the 95^th^ percentile (>1%–6%), and this occurred primarily in individuals under 30 years of age, where 7.5% had values above normality.

**Table 3:** Correlation with age for bronchoalveolar lavage fluid findings in healthy never-smoking subjects.

Variable	N	Pearson Correlation Coefficient	Prob>|r| under H0: Rho = 0
**Return volume** (mL)	266	−0.30737	<.0001
**Recovery** (%)	266	−0.30405	<.0001
**Viability** (%)	292	0.02744	0.6405
**Total Cell Number** (×10^6^)	266	−0.02816	0.6475
**Cell concentration** (×10^6^/L)	266	0.09078	0.1398
**Macrophages** (%)	255	0.11186	0.0746
**Macrophages** (×10^6^/L)	284	0.00588	0.9214
**Lymphocytes** (%)	255	0.02524	0.6883
**Lymphocytes** (×10^6^/L)	284	0.00247	0.9669
**Neutrophils** (%)	255	0.04229	0.5014
**Neutrophils** (×10^6^/L)	284	0.01233	0.8360
**Eosinophils** (%)	254	−0.10705	0.0887
**Eosinophils** (×10^6^/L)	283	−0.11679	0.0497
**Basophils** (%)	255	−0.05543	0.3780
**Basophil** (×10^6^/L)	284	−0.02549	0.6688
**Mast Cells** (per 10 visual fields)	214	−0.03260	0.6354

No gender related correlations where seen for any of the BALF analyses parameters (Table 4, concentrations of cell subgroups not shown).

**Table 4:** Comparison of bronchoalveolar lavage fluid findings between healthy never-smoking females and males.

	Females			Males			
Variable	N	Mean	Std Dev	N	Mean	Std Dev	P
**Age** (years)	163	31.4	11.5	132	31.7	11.9	0.797
**Return volume** (mL)	149	181.1	23.3	117	178.1	24.0	0.313
**Recovery** (%)	149	72.3	9.3	117	71.3	9.6	0.374
**Viability** (%)	160	91.8	4.9	132	91.6	5.2	0.766
**Total Cell Number** (×10^6^)	149	16.1	8.2	117	16.6	6.9	0.614
**Cell concentration** (×10^6^/L)	149	89.7	43.1	117	94.7	39.9	0.334
**Macrophages** (%)	156	88.4	8.4	128	87.6	8.0	0.373
**Lymphocytes** (%)	156	9.38	8.02	128	9.98	7.31	0.517
**Neutrophils** (%)	156	1.69	1.30	128	2.05	2.53	0.147
**Eosinophils** (%)	155	0.25	0.44	128	0.33	0.80	0.300
**Basophils** (%)	156	0.02	0.07	128	0.02	0.06	0.658
**Mast Cells** (per 10 visual fields)	113	2.40	3.12	101	3.59	6.10	0.078

### Intra-individual variability of bronchoalveolar lavage findings

**Figure 2:**
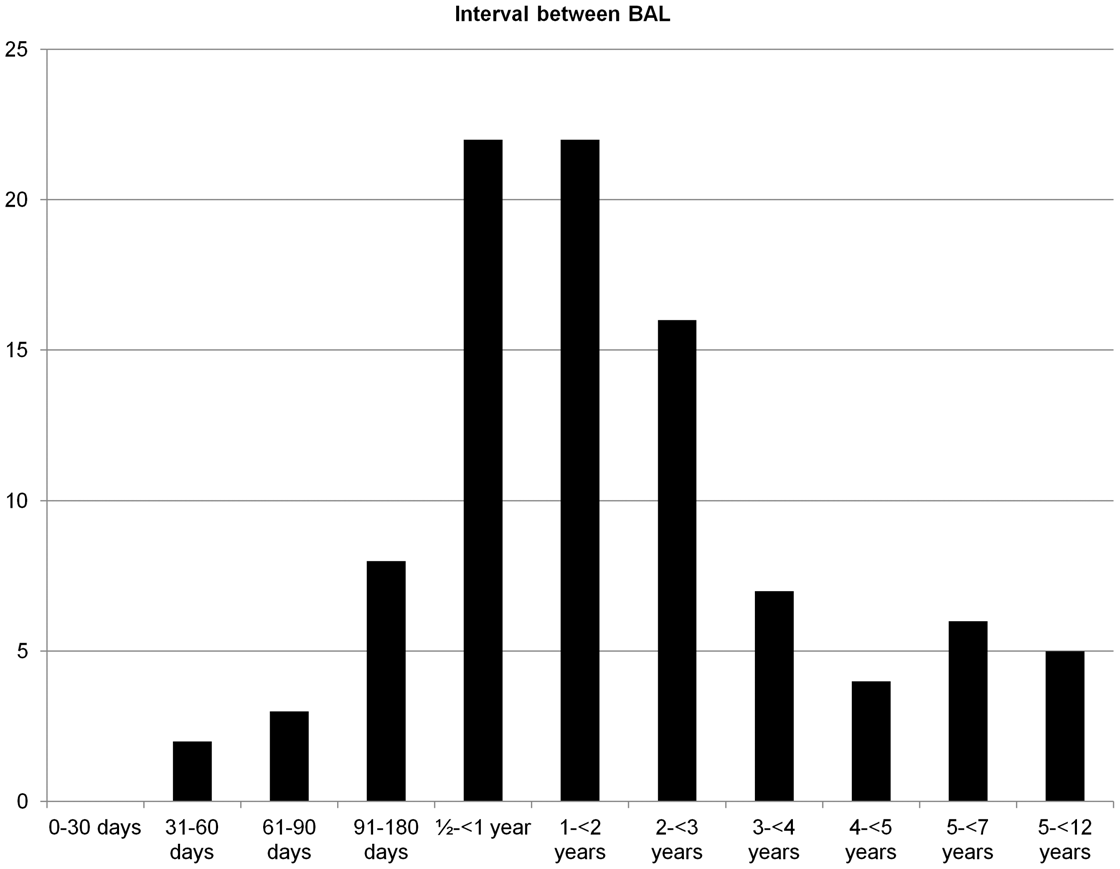
Interval between bronchoalveolar lavages in healthy never-smoking subjects undergoing more than one investigation (number of investigations on y-axis).

Forty seven volunteers had 2–5 repeat lavages. The shortest time period in between lavages was one month and the longest was 12 years (Figure 2).

No significant differences were found between the first and the repeat lavages for any of the BALF parameters. However, a relatively large individual fluctuation was present, mainly for macrophages and lymphocytes. The percentage of macrophages demonstrated the largest normal variability (10.2%), whereas the lymphocytes had a normal variability of 9.2% (Table 5).

**Table 5:** Intraindividual variability of bronchoalveolar lavage fluid findings in healthy never-smoking subjects.

Variable	N	Std Dev	Normal variability
**Return volume** (mL)	43	15.80	30.97
**Recovery** (%)	43	6.38	12.50
**Viability** (%)	47	3.71	7.27
**Total Cell Number** (×10^6^)	43	4.67	9.15
**Cell concentration** (×10^6^/L)	43	24.47	47.97
**Macrophages** (%)	47	5.22	10.24
**Lymphocytes** (%)	47	4.74	9.28
**Neutrophils** (%)	47	0.82	1.60
**Eosinophils** (%)	47	0.20	0.39
**Basophils** (%)	47	0.04	0.07
**Mast Cells** (per 10 visual fields)	28	2.60	5.10

**Figure 3:**
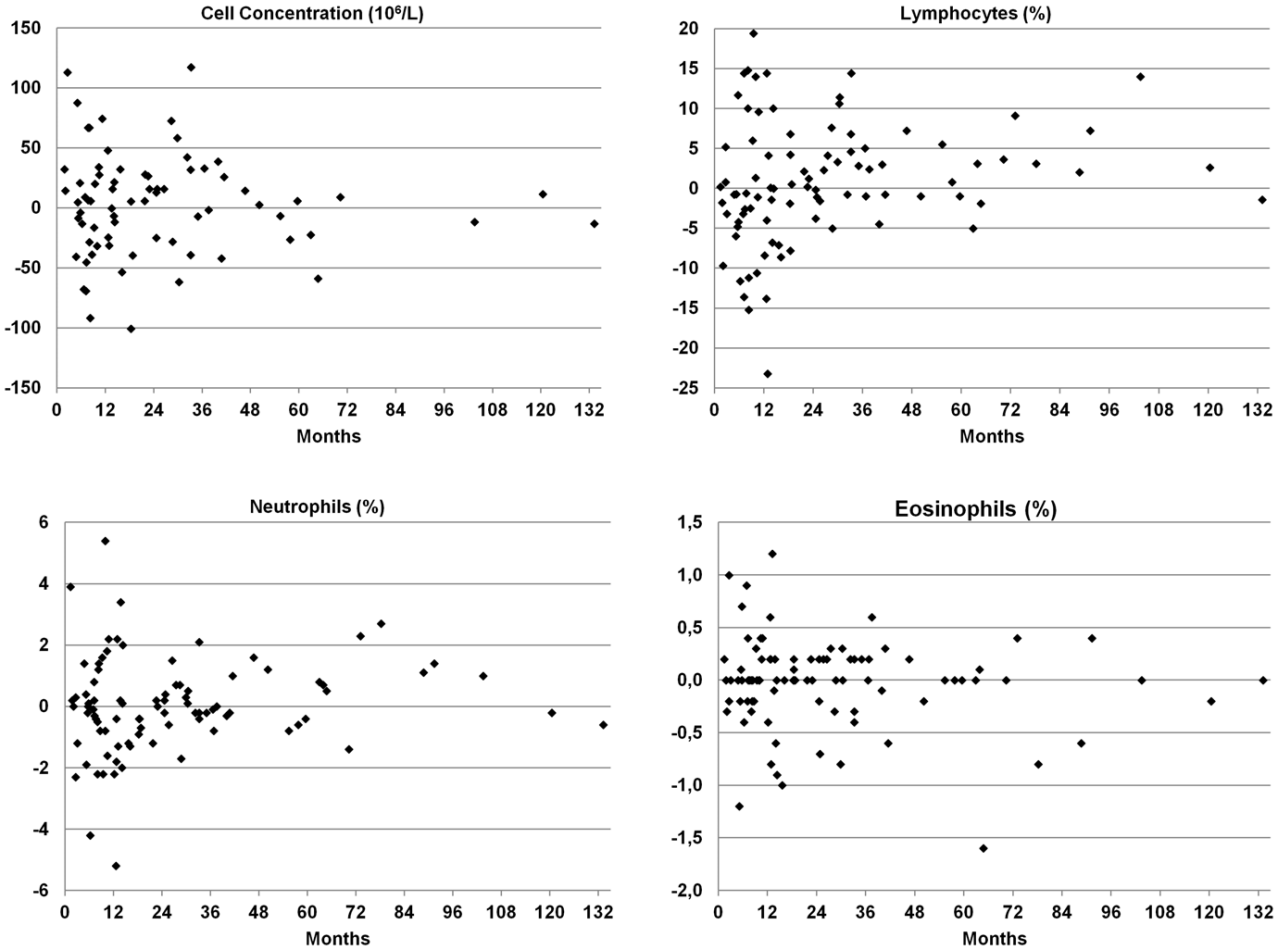
Change in bronchoalveolar lavage fluid findings from first BAL (y-axis) related to time between investigations (x-axis) in healthy never-smoking subjects.

There was no correlation between BALF results and the chronological number of the bronchoscopy, nor was there any correlation with the time period in-between lavages (Figure 3).

### Comparison of BALF parameters between the middle lobe and the lingula, and BALF during the calendar year

There was no difference in the BAF results whether the fluid was collected from the lingula or the middle lobe (Table 6).

**Table 6:** Comparison of bronchoalveolar lavage fluid findings between the middle lobe and the lingula in healthy never-smoking subjects.

	Middle lobe			Lingula			
Variable	N	Mean	Std Dev	N	Mean	Std Dev	p
**Age** (years)	281	31.9	11.8	14	24.9	4.4	<.0001
**Return volume** (mL)	252	179.6	23.9	14	182.3	18.2	0.6824
**Recovery** (%)	252	71.8	9.5	14	72.9	7.2	0.6926
**Viability** (%)	278	91.6	5.1	14	94.1	4.0	0.0667
**Total Cell Number** (×10^6^)	252	16.4	7.8	14	14.6	4.1	0.1443
**Cell concentration** (×10^6^/L)	252	92.5	42.4	14	80.9	23.9	0.1099
**Macrophages** (%)	270	88.1	8.3	14	87.4	5.9	0.7540
**Lymphocytes** (%)	270	9.60	7.80	14	10.55	5.42	0.6540
**Neutrophils** (%)	270	1.86	2.00	14	1.57	0.69	0.2015
**Eosinophils** (%)	269	0.28	0.63	14	0.46	0.57	0.2982
**Basophils** (%)	270	0.02	0.06	14	0.01	0.05	0.7772
**Mast Cells** (per 10 visual fields)	200	2.83	4.54	14	4.93	7.50	0.3180

**Figure 4:**
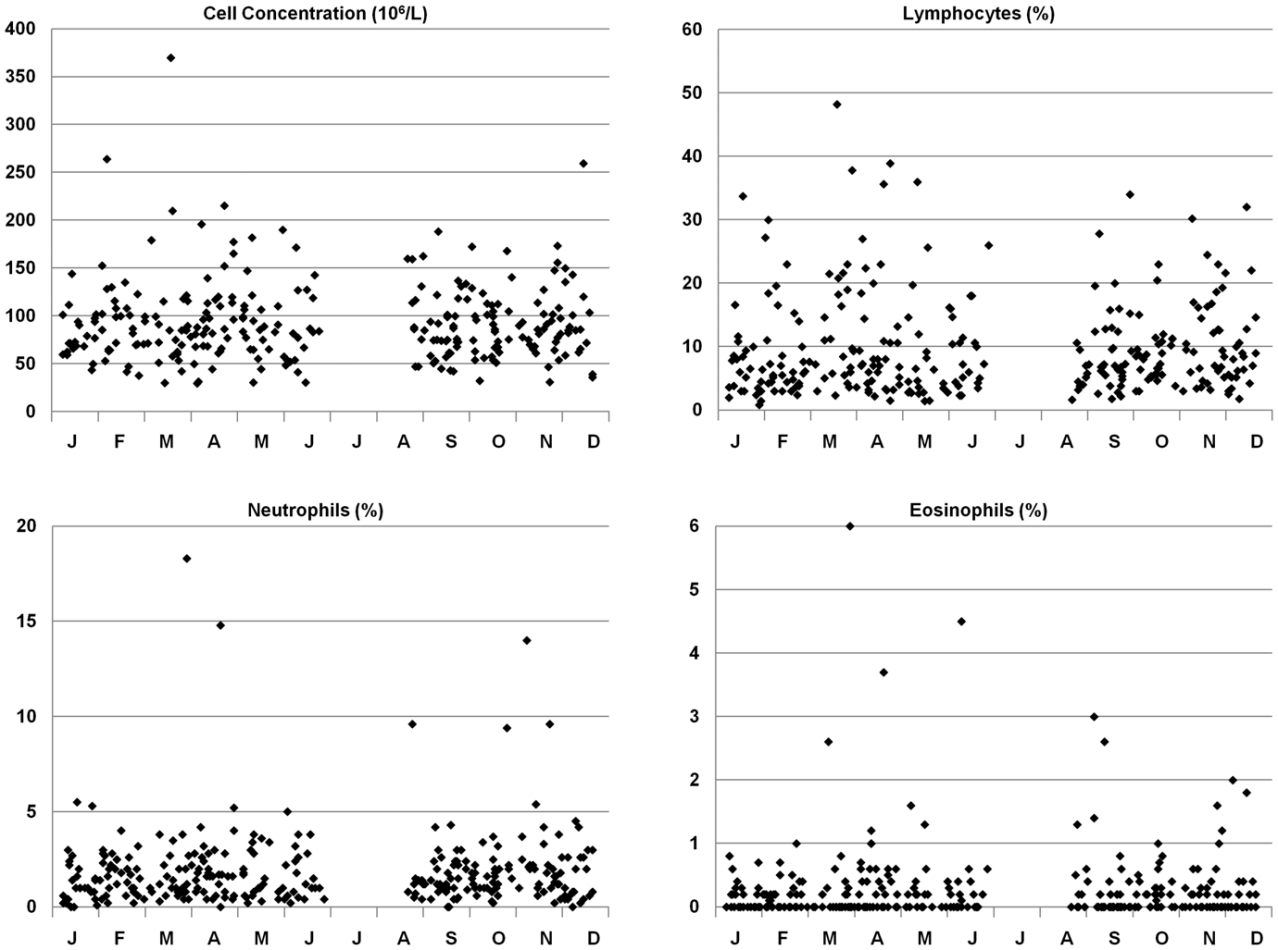
Bronchoalveolar lavage fluid findings (y-axis) during the calendar month (x-axis) in healthy never-smoking subjects.

Furthermore, there was no seasonal variation in BALF results (Figure 4).

## Discussion

This retrospective analysis of BALF data from 295 healthy never-smoking volunteers investigates the influence of age, gender, collection site and season on bronchoalveolar lavage cellularity. The results demonstrate an age dependent decrease of lavage fluid return, but other than that, the BALF cell parameters were independent of age, gender, season and collection site (lingula vs. middle lobe). The 5^th^ to the 95^th^ percentile for the BALF cell differential count was 72–96% for macrophages, 2–26% for lymphocytes, 0–4% for neutrophils and 0–1% for eosinophils. Basophils and mast cells were rare. When repeat lavages were performed, there was a relatively large intra-individual variability, mainly for macrophages and lymphocytes.

The present study is, to our knowledge, the largest single-centre study on BALF composition in healthy volunteers to date, and is statistically well-powered. Furthermore, it is the only study so far to adequately address reference values for BALF composition in individual subgroups. There are no major concerns about methodological differences as bronchoscopies were performed by a limited number of experienced investigators and the analysis of the BALF followed the same standardized procedure throughout. Previous studies were limited by small sample sizes and methodological differences across trials and the majority of participants were males <50 years of age. Thus, the results from those studies should not be extrapolated to cover other subgroups, such as women and older individuals.

Furthermore, in previous studies reference values were defined as mean ±2 SD. However, this is an approximation assuming a normal distribution, which is not the case for several of the BALF parameters. The percentiles reflect the actual observed value, and thus we have chosen to use the 5^th^–95^th^ percentile as cut off points. This gives a somewhat narrower definition of normality, as only 90% of the values will fall within the normal range.

A guideline on bronchoalveolar lavage cellular analysis was recently published by the ATS [18]. This guideline, taking into account 7 published studies, established normal values as >85% for alveolar macrophages, 10–15% for lymphocytes, ≤3% for neutrophils, ≤1% for eosinophils and mast cells ≤0.5%. Although, a majority of our healthy volunteers did have cell differential counts that would fall within these, and other, previously described reference values, there was a large inter-individual variability. Particularly for macrophages and lymphocytes the fluctuation between subjects was large and approximately 10% had a percentage of lymphocytes over 20%. The patient's medical journals did not provide any obvious explanation for the disproportionate values, and they must therefore be considered a variation of normality. Similarly, 10% of subjects had a macrophages under 76%. Thus, a rather large proportion of our normal volunteer's would fall outside the ATS definition of BAL cellular patterns in normal adult non-smokers. In agreement with other investigators [10], [11] we found an age dependent decrease of lavage fluid return. This can probably be explained by the loss of elastic recoil seen in older individuals resulting in an obstructive lung function pattern [25], [26]. This hypothesis is supported by previous findings that BALF recovery in smokers and patients with chronic obstructive pulmonary disease (COPD) is dependent on the extent of emphysema on high resolution computered tomography (HRCT) [27]. We did not, however, find a correlation between age and increased levels of lymphocytes and/or neutrophils. This may be explained by the fact that, while other investigators have described age dependent differences in individuals up to 83 years of age, the oldest individuals included in our material were 65. Furthermore, the number of older individuals included in previous trials has been very small, and thus the results must be interpreted with caution. The high eosinophil counts, although relatively few, are interesting as they were seen primarily in individuals under the age of 30. This indicates that in younger adults, a raised eosinophil count (>6%) can be a variation of normality.

Our study did not demonstrate any correlation between gender and BALF cell differential count. Also, fluid recovery was similar in both groups, despite that males have larger lung volumes. The subjects were well matched for age and there was an almost equal distribution between groups (female individuals were slightly overrepresented). Therefore, any real difference between the two sexes would have been evident.

The large group of female volunteers included in our study is unique, and our results justify the use of the same reference values for male and female individuals.

All volunteers lived in an urban environment in the greater Stockholm area. Certain data indicate that exposure to air pollutants may cause higher numbers of BAL fluid total cell counts, lymphocytes and alveolar macrophages [28], neutrophils [29], eosinophils [30] and/or CD 8 positive T-lymphocytes [31]. The significance of these rather disparate findings cannot be established at present. However, it is possible that the reference values obtained in this study may not be fully applicable to a rural population.

The intra-individual variability in our study was relatively large, but was independent of the number or sequence of the BAL. Furthermore the median values for BALF differential cell counts within subjects were consistent over time. These findings suggests that isolated higher values of one or other parameter may occur without evidence of disease, but these values outside the reference limits rarely persist over time. As the time intervals between bronchoscopies were generally large, no conclusion can be made regarding the intra-individual variability of BALF differential cell count over a short time period.

Previous studies have shown a change in BALF composition after exposing healthy individuals to cold air [32]. We hypothesized that this may translate into a seasonal variation in BALF results. Our data show, however, that this is not the case, indicating that bronchoscopy with BAL could be performed independent of calendar month in healthy controls.

In conclusion, the present study is to our knowledge the largest single-centre study on BALF composition in healthy volunteers to date, and individual subgroups are well represented. Provided that the same methodology is used for collection and analysis of BALF, our results may provide reference values for use in clinical practice. There was no correlation between cytospin cell differential counts and age, gender or season. Thus, it seems acceptable to use the same reference values for all never-smoking individuals aged 65 or less. Furthermore, BALF results do not seem to be affected by the collection site (lingula or middle lobe), suggesting that BAL could be performed in the lobe easiest to access. The largest intra- and inter-individual variability for the cytospin cell differential counts was seen for macrophages and lymphocytes, resulting in wider reference values than those previously reported.

Limitations: The majority of subjects included in our study were of Scandinavian origin, living in an urban environment, raising the concern that the results may not be fully applicable to other populations. However, our results do not greatly differ from those reported in other trials, suggesting that there are no major differences in the BALF composition at least for the European and the North American populations. Furthermore, no subjects over the age of 65 years were included in the study, and the results may not be applicable to the elderly population.
